# Dust Vortex in the Taklimakan Desert by Himawari-8 High Frequency and Resolution Observation

**DOI:** 10.1038/s41598-018-37861-4

**Published:** 2019-02-04

**Authors:** Keiya Yumimoto, Mizuo Kajino, Taichu Y. Tanaka, Itsushi Uno

**Affiliations:** 1 Research Institute for Applied Mechanics, Kyushu University, Kasuga, Fukuoka 816–8580 Japan; 2 Meteorological Research Institute, Tsukuba, Ibaraki 305–0052 Japan; 3 Faculty of Life and Environmental Sciences, University of Tsukuba, Tsukuba, Ibaraki 305–8577 Japan

## Abstract

The Taklimakan Desert is known to be one of the world’s major sources of aeolian dust particles. Continuous images with 10-min temporal and 2-km spatial resolutions from a new-generation geostationary meteorological satellite captured the lifecycle (generation, evolution and outflow) of a previously unrecognized type of Taklimakan dust storm. The dust storm showed an anti-clockwise spiral structure and a clear core and behaved like a “dust vortex”. From image analysis, the horizontal scale and temporal lifetime of the dust vortex were estimated to be 600 km and 36 hours, respectively. We found that a strong pressure trough (cut-off low), along with a cold air mass located on the northwestern side of the Taklimakan Desert and the high mountains surrounding the Taklimakan Desert, played important roles in the formation and evolution of the dust vortex.

## Introduction

Dust particles from the Taklimakan Desert (hereafter, TD) are transported beyond the Pacific Ocean, sometimes undergoing more than one full circuit around the globe^1^. Unlike the Gobi Desert, which is spread over the relatively flat ground, the TD is located in the Tarim Basin and extends for ~1500 km east to west and ~550 km north to south, with an area of ~320,000 km^2^. It is surrounded on three sides (north, west, and south) by mountains exceeding the altitude of 5000 m (mean sea level (MSL)), including the Tianshan Mountains, Pamir Plateau, and Kunlun Mountains, and has an open area (the eastern corridor) on the eastern side (Fig. S1). This unusual and complex terrain is closely related to the outbreak, entrainment, and transport of dust particles from the TD.

The generation of dust storms in the TD was previously studied using ground- and space-based observations and numerical models. A 40-year dust storm record indicated that dust storms were mainly associated with the easterly cold airflow that flows into the TD through the eastern corridor^2^. A numerical simulation using a mesoscale meteorological model suggested that the large-scale flow associated with the surface low-pressure system strongly affected the development of the mesoscale wind flow that caused dust outbreaks on 12–15 April 2002^3^. A dust transport installed in a nested regional meteorological model reproduced the dust emissions associated with the complicated wind flows, including strong down-slope winds from the Tianshan Mountains and a strong easterly wind flow from the eastern corridor^4^. Comprehensive analysis using satellite observation and numerical models showed that the unusual terrain combined with the strong easterly wind in the eastern corridor enabled massive amounts of dust to be injected into the upper atmosphere, which were then transported eastward beyond the Pacific Ocean^5,6^. The meteorological conditions that cause dust storms in the TD were investigated using ground-based data, meteorological reanalysis products, and mesoscale numerical simulations and classified into three patterns^7,8^ (for more details, see supplementary information and Fig. S2). All dust storms referred to in this paragraph fell under the patterns.

The Advanced Himawari Imager (AHI) onboard Himawari-8, a new-generation geostationary meteorological satellite (GMS) put into operation on July 7, 2015, has 16 observational bands from visible to infrared, with unprecedented spatial (0.5–1.0 km for visible and 1–2 km for infrared) and temporal (10-minute) resolution covering wide areas of the globe (East and Southeast Asia, the western Pacific Ocean, Oceania, and the Australian continent)^9^. From 30 April to 1 May 2017, continuous images from AHI captured details of a dust storm with a spiral structure in the TD. The dust storm had a clear core and horizontal and temporal scales of 600 km and 36 hours. To the best of our knowledge, this type of dust phenomenon has not been previously reported, and the meteorological conditions that caused the dust storm have escaped classification. In this study, we investigated the generation and evolution of the dust storm using observations near the surface and from space and through model simulation with a regional-scale meteorology–chemistry model.

## Results

**Figure 1:**
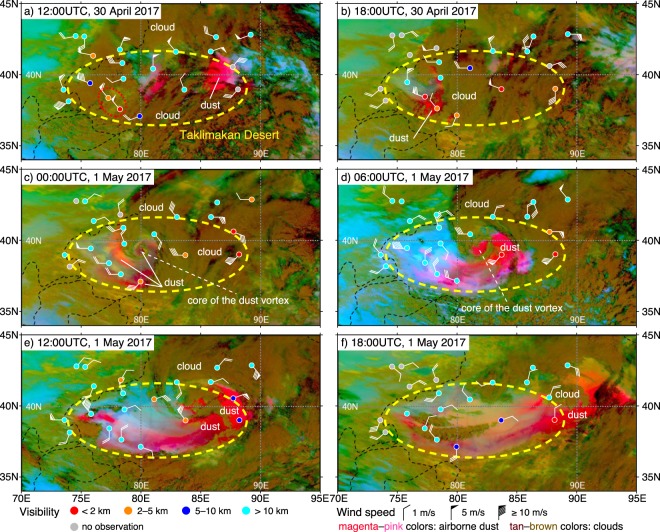
Dust RGB imagery (from supplementary movie (Movie S1)) and the SYNOP-observed visibility and wind speed over the Taklimakan Desert during the dust vortex. In the Dust RGB imagery, airborne dust and clouds are depicted in magenta–pink and tan–brown colors, respectively. The Dust RGB images were obtained from “Himawari Real-Time Image/Central Asia/Dust RGB” (Meteorological Satellite Center of Japan Meteorological Agency) (http://www.data.jma.go.jp/mscweb/data/himawari/).

The continuous images at 10-min intervals from AHI clearly captured the lifecycle (generation, evolution and outflow) of the dust storm that occurred in the TD from 30 April to 1 May 2017 (Movie S1). Figure 1 shows snapshots from the movie of the generation, evolution and outflow periods. In its generation period, strong northwesterly winds of 5–9 m/s and the outbreak of the dust storm were observed in the western part of the TD (Fig. 1a,b). Then, during its evolution, the dust storm formed an anti-clockwise spiral structure in the central part of the TD, and its core was clearly identified in the center of the dust storm in the images from AHI (Fig. 1c,d). On the following day, the dust storm advanced to the east, and its spiral structure was broken around the eastern corridor (Fig. 1e,f). The horizontal scale (diameter) and lifetime of the dust storm were estimated to be approximately 600 km and 36 hours (from 6 UTC on 30 April to 18 UTC on 1 May), respectively (Movie S1). Our team named this dust phenomenon a “dust vortex” to emphasize its spiral structure and horizontal scale. Images from earth observation satellites in polar orbit (MODerate-resolution Imaging Spectroradiometer) also detected the occurrence of the dust vortex (Fig. S3). However, it was difficult to obtain an overview of the generation and evolution of such a short-temporal scale phenomenon from once-daily observations. The quasi-continuous observations from the GMS overcame this temporal limitation.

**Figure 2:**
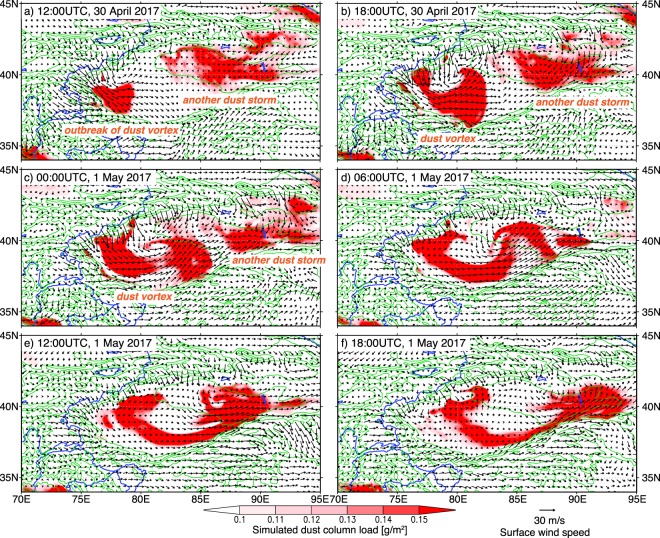
Simulated dust column load (magenta) and surface wind (black vectors) with the topography (green lines) from the supplementary movie (Movie S2). The illustrated region and period are the same as those in Fig. 1. Wind vectors are shown at 5 model-grid intervals.

**Figure 3:**
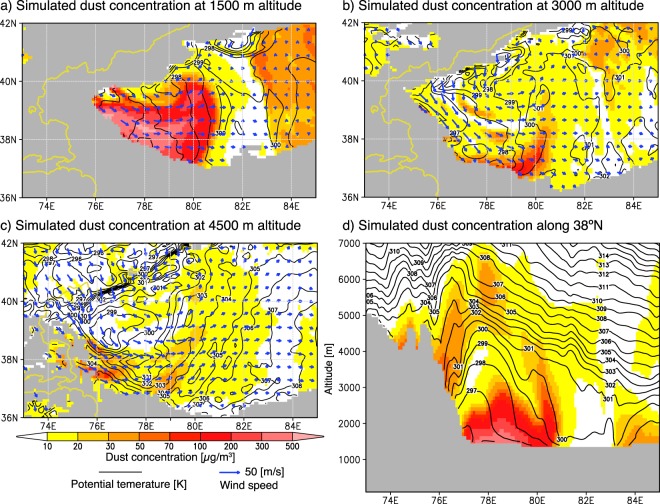
Simulated dust concentrations (color), potential temperatures (black solid lines), and wind fields (blue vectors) at 15 UTC on 30 April 2017. (**a**–**c**) Horizontal distributions at 1500, 3000, and 4500 m MSL. (**d**) Cross-section along 38**°**N.

The model simulation successfully reproduced the generation and evolution of the dust vortex observed by AHI and the complicated wind fields that caused the dust vortex (Movie S2). In the generation period, a strong northwesterly wind blew into the TD through the boundary between the Tianshan Mountains and the Pamir Plateau (around 40°N and 75°E) and suspended a large amount of dust in the air (Fig. 2a,b). The simulated northwesterly wind was consistent with the observed wind (compare Figs 1a and 2a as well as 1b and 2b). Figure 3 shows the simulated 3-dimensional structure of the dust vortex at 15 UTC on 30 April (between Fig. 2a,b). The low potential temperature that spread out in the shape of a fan over the western part of the TD indicates that the strong northwesterly wind was driven by the incursion of a cold air mass through the boundary between the Tianshan Mountains and the Pamir Plateau (Fig. 3a). The cold airflow had the characteristics of a gravity current and formed a cold front. The thickness of the front was approximately 3000–4000 m, and the uplifted dust (>10 µg/m^3^) was entrained to an elevation of ~7000 m MSL at the front of the cold front (Fig. 3b–d), where it could contribute to a longer atmospheric lifetime than the dust from the Gobi Desert^10^, atmospheric background aerosol^11^, and long-term transport^5,6^. Tan–brown colors (i.e., clouds) indicate areas where NHM-Chem predicted the occurrence of the dust vortex (Figs 1a and 2a), and the SYNOP stations indicated by red circles in Fig. 1a reported “dust or sand raised by wind (ww = 7)” as the present weather condition. This indicates that the outbreak occurred under clouds and could not be detected in the images from space. Another dust storm, which was caused by an easterly wind intruding through the eastern corridor before the outbreak of the dust vortex, was captured by both the Dust RGB image and a numerical simulation of the eastern side of the TD (Figs 1a and 2a).

In the evolution period, forced by the mountains along the southern side of the TD (the Kunlun Mountains), the northwesterly wind changed its direction to westerly-southwesterly (Fig. 2b,c). At the same time, a northerly wind moved into the TD, crossing over the Tianshan Mountains (the northern side of the TD). The western part of the northerly wind (75–80°E) boosted the westerly-southwesterly wind, while the eastern part (80–85°E) encountered the front of the westerly-southwesterly wind, changed its direction to easterly, and eventually formed the vortex structure and its core (Fig. 2c,d). Figure S4 in the Supporting Information shows a comparison of the cross-section of the simulated dust concentration and the total attenuated backscatter observed by a space-borne lidar (Cloud-Aerosol Lidar and Infrared Pathfinder Satellite Observation (CALIPSO)) during the evolution period (21 UTC 30 April). The simulated dust showed good agreement with the observations (e.g., the depth of the dust layer and the elevated dust layer above the northern slope) (Fig. S4a and b). A meridional cross-section of the dust concentration and zonal wind showed that although the 1-km deep dust layer covered all of the TD, the wind direction was opposite to that in the northern and southern parts of the TD (Fig. S4b). The spiral structure of the dust and wind field reached an altitude of ~6000 m MSL (the core of the vortex was around 40°N and 80°E) (Fig. S4b–d).

On the following day, the dust vortex advanced to the east and came to an end (Fig. 2e,f). Toward the eastern corridor, the north-south width of the TD became narrower. Advancing eastward, the dust vortex was suppressed by steep mountains on the northern and southern sides of the TD (the Tianshan and Kunlun Mountains), the spiral structure became difficult to sustain, and eventually, the vortex diminished. The uplifted dust was transported outside of the TD through the eastern corridor.

## Discussion

**Figure 4:**
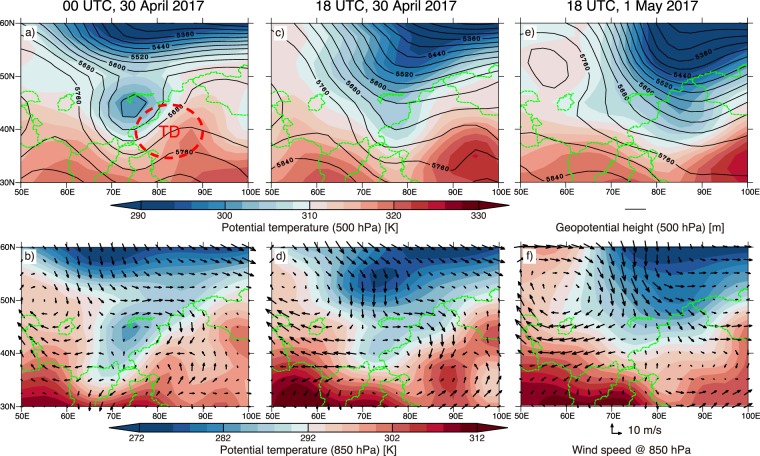
Meteorological variables around the Taklimakan Desert at (**a**,**b**) 00 UTC on 30 April, (**c**,**d**) 18 UTC on 30 April, and (**e**,**f**) 18 UTC on 1 May from the NCEP/NCAR reanalysis data. The upper panels show potential temperatures (colors) and geopotential heights (black solid lines) at 500 hPa. The lower panels show potential temperatures (colors) and wind velocities (vectors) at 850 hPa.

We investigated the meteorological conditions surrounding the generation and evolution of the dust vortex using meteorological reanalysis data and found that a strong pressure trough (cut-off low) that advanced in the northwestern side of the TD characterized the meteorological conditions (Fig. 4). Before the outbreak of the dust vortex, the cut-off low of 500 hPa and its associated cold air mass were located near Lake Balkhash (northwestern side of the TD) (Fig. 4a). At 850 hPa, a strong potential temperature gradient was found along the Pamir Plateau and Tianshan Mountains (western and northern sides of the TD) (Fig. 4b). During the generation and evolution of the vortex, the trough merged with the cold low and gradually advanced eastward (Fig. 4c). The cold air mass accompanied by the trough crossed over the Pamir Plateau and Tianshan Mountains and generated strong northwesterly and northerly winds in the TD (Fig. 4d). However, the cold front and the spiral structure shown in the images from AHI and the model simulation were not found in the meteorological reanalysis data due to its coarse horizontal resolution. The horizontal resolution of 2.5° × 2.5° was too coarse to represent the high, steep mountainside terrain surrounding the TD. On the following day, the trough moved further to the east (Fig. 4e). The cold air mass on the western side of the TD and the westerly wind across the Pamir Plateau disappeared (Fig. 4f). Taken together with the results of the model simulation, we can conclude that the strong trough (cut-off low) with a cold air mass located on the northwestern side of the TD drove the strong northwesterly and northerly winds, crossing over the Pamir Plateau and Tianshan Mountains, and that the steep mountainside along the southern side of the TD (the Kunlun Mountains and Tibetan Plateau) played a key role in the generation and evolution of the dust vortex (Fig. S2d).

Meteorological conditions and wind fields that cause dust storms in the TD were previously studied and classified into three patterns^7,8^ (for more details, see supplementary information and Fig. S2). We compared those that caused the dust vortex with those of the three patterns. The location and advancement path of the trough and the generation of the northwesterly wind that induced the dust vortex resembled Pattern 3, in which a cold trough moves eastward over the Pamir Plateau, and a cold air mass then flows into the TD through the boundary between the Tianshan Mountains and the Pamir Plateau (Fig. S2c). However, the trough in Pattern 3 is weaker than that observed in the dust vortex, and the horizontal scale of dust storms in Pattern 3 is relatively small compared with that of the dust vortex. In Pattern 3, it was concluded that strong westerly winds and dust storms are generated only in the western and southwestern parts of the TD. Moreover, Pattern 3 is not characterized by a strong northerly wind across the Tianshan Mountains, which had an important role in the formation of the spiral structure of the dust vortex. The strong northerly wind across the Tianshan Mountains is classified into Pattern 2, in which a trough near the northern side of the Tianshan Mountains advances southeastward, approaching the northern side of the Tianshan Mountains, and the cold air accompanied by the trough flows directly across the Tianshan Mountains (Fig. S2b). Although Pattern 2 frequently produces very strong wind and has caused the largest dust storm out of the three patterns in the wide area of the TD, the northwesterly wind that drove the outbreak of the dust vortex is not generated in Pattern 2. The location and path of the trough in the dust vortex followed a different pattern from those in Pattern 2. We concluded that the meteorological conditions (the location and moving path of the trough) that caused the dust vortex resembled those of Pattern 3; however, the stronger trough (cut-off low) drove the strong northerly wind across the Tianshan Mountains and differentiated the dust vortex from the dust storms classified in Pattern 3.

To estimate the frequency of the occurrence of dust vortexes in the TD, we reviewed the Dust RGB images from Himawari-8 from 1 September 2016 to 12 April 2018 and found another dust vortex on 13–14 April 2017 (Movie S3 in the Supporting Information) (i.e., dust phenomena classified in the dust vortex were caused at least twice in 19 months). The meteorological conditions around the TD during 13–14 April 2017 also showed that the cold air mass associated with the strong trough located on the northwest side of the TD triggered the formation of the dust vortex (Fig. S5). The core and spiral structure of this dust vortex were less obvious than those of the event analyzed in this study (compare Movies S1 and S3 in the Supporting Information). The warmer cold air mass and weaker trough on 13–14 April 2017 compared to that on 30 April–1 May 2017 can explain this difference (compare Figs 4 and S5).

## Methods

### Dust RGB image from Himawari-8

We used Dust RGB images derived from composite data from the Advanced Himawari Imager onboard Himawari-8, a Japanese third-generation GMS^9^. Dust RGB images leverage the different optical characteristics (absorption and scattering) in the infrared wavelengths (8.6, 10.4, and 12.4 µm) between dust and clouds^12,13^. In Dust RGB images, airborne dust and clouds are depicted in magenta–pink and tan–brown colors, respectively. Unlike other aerosol optical properties retrieved from measurements in the visible wavelength (e.g., aerosol optical thickness), Dust RGB images are available during both day and night, obtained every 10 minutes with 2-km horizontal resolution. Dust RGB images were previously used to analyze the occurrence and transport of large-scale dust storms in the Gobi Desert^14^.

### Meteorological data

The wind speed, wind direction, visibility, and weather reported in the surface synoptic observations (SYNOP) data^15^ were referred to as wind fields and used as a proxy of dust concentrations in and around the TD. We used meteorological reanalysis data provided by the National Center for Environmental Prediction (NCEP) and the National Center for Atmospheric Research (NCAR)^16^ to investigate meteorological conditions around the TD.

### Numerical simulation

NHM-Chem is a 3D regional-scale meteorology–chemistry model^17^. We used the bulk equilibrium method, in which aerosols are classified into three categories (submicron, dust, and sea salt) to investigate the detailed meteorological conditions and reproduce the mesoscale dust vortex phenomena in the TD. Dust emission fluxes can be predicted as a function of the friction velocity^18^, taking surface conditions (e.g., snow and vegetation coverage) into account. The emission, transport, and deposition of simulated Asian dust have been evaluated by regional-scale chemical and physical *in situ* observation data, as presented^19,20^. In this study, we employed the total concentration of dust particles with diameters of less than 10 µm as the simulated dust concentration. The model domain (see Fig. S1 in Supporting Information) was centered at 40°N and 83°E, with a horizontal resolution of 9 km and 404 × 284 grid points. The top of the model atmosphere was 18 km. There were 41 vertical layers, with the thickness gradually increasing in the upper layers. JRA-55 global reanalysis^21^ was used as the initial and boundary conditions.

## Supplementary information

Supplementary info

Movie S1

Movie S2

Movie S3

## Data Availability

The datasets generated during the current study are available from the corresponding author on reasonable request.
