# Supplementary material for: Dust Vortex in the Taklimakan Desert by Himawari-8 High Frequency and Resolution Observation
Source: Sci Rep. 2019 Feb 4;9:1209. doi: 10.1038/s41598-018-37861-4 (PMC6362108; doi:10.1038/s41598-018-37861-4)
Supplement: Supplementary file 1 — Supplementary info [file 41598_2018_37861_MOESM1_ESM.docx]

**Supplementary information for “Dust Vortex in the Taklimakan Desert by Himawari-8 High Frequency and Resolution Observation”**

Keiya YUMIMOTO^1,2,*^, Mizuo KAJINO^2,3^, Taichu Y. TANAKA^2^, Itsushi UNO^1^

^1^Research Institute for Applied Mechanics, Kyushu University, Kasuga, Fukuoka, 816-8580, Japan

^2^Meteorological Research Institute, Tsukuba, Ibaraki, 305-8577, Japan

^3^Faculty of Life and Environmental Sciences, University of Tsukuba, Tsukuba*,* Ibaraki, 305-0006, Japan

*yumimoto@riam.kyushu-u.ac.jp

**Meteorological conditions that caused dust storms in the Taklimakan Desert**

In the Taklimakan Desert (TD), dust storms are generated by a mesoscale cold wind system, and the meteorological conditions that generate dust storms can be classified into three patterns as follows^1,2^.

**Pattern 1: easterly wind (Fig. S2a).** A trough located near the northeastern side of the TianShan Mountains gradually advances southeastward. The cold westerly wind accompanied by the trough changes its direction to westward after going around the eastern corridor (an open area along the eastern side of the TD) and flows into the TD as a cold easterly wind. At first, the cold easterly wind blows dust particles up in the eastern part of the TD. Then, as the front of the easterly wind advances westward, dust storms occur in the area extending to the west, and finally, all of the TD is covered by the airborne dust.

**Pattern 2: northerly wind (Fig. S2b).** A trough located near the northern side of the TianShan Mountains (more westerly compared with the Pattern 1) advances southeastward, approaching the northern side of the TianShan Mountains. The cold air accompanied by the trough flows directly across the TianShan Mountains. The strong northly wind spreads from the southern foot of the TianShan Mountains to the southern part of the TD, generating dust storms in the wide area of the TD. Pattern 2 frequently produces a very strong wind in the TD and consequently, has caused the largest dust storm out of the three patterns.

**Pattern 3: easterly cold wind (Fig. S2c).** As a relatively weak trough advances eastward, the cold air mass flows into the TD through the boundary between the TianShan Mountains and the Pamir Plateau, and dust storms are generated only in the western and southwestern parts of the TD. The magnitudes of the dust storms are usually small compared with those of storms produced by the two other patterns.

**Satellite data**

**Aerosol optical depth from the Moderate-Resolution Imaging Spectroradiometer (MODIS).** MODIS is a visible and infrared image sensor onboard the Terra and Aqua satellites. We used aerosol optiocal depth (AOD) retrieved by the Deep Blue algorithm^3^ in the Level 2 correction 6 MODIS aerosol product (MOD04_L2.006 and MYD04_L2.006).

**Total attenuated backscatter from Cloud-Aerosol Lidar with Orthogonal Polarization (CALIOP) aboard the Cloud-Aerosol Lidar and Infrared Pathfinder Satellite Observation (CALIPSO).** CALIOP, onboard CALIPSO, is a space-borne lidar^4^. We used the 532 nm total attenuated backscatter provided by the CALIPSO Lidar Level 1B profile data, standard version 4.10 (CAL_LID_L1-Standard-V4-10).

**References**

1. Aoki, I., Kurosaki, Y., Osada, R., Sato, T. & Kimura, F. Dust storms generated by mesoscale cold fronts in the Tarim Basin, Northwest China. *Geophys. Res. Lett.* **32,** L06807 (2005).

2. Aoki, I. The process of dust storm generation in Tarim Basin, Northwest China. *Master Thesis of Institute of Geoscience, Tsukuba University* (2003) in Japanese.

3. Hsu, N., Tsay, S. & King, M. Deep blue retrievals of Asian aerosol properties during ACE-Asia. *Geosci. Remote* **44,** 3180–3195 (2006).

4. Winker, D. M. *et al.* The CALIPSO Mission: A Global 3D View of Aerosols and Clouds. *Bull. Am. Meteorol. Soc.* **91,** 1211–1229 (2010).


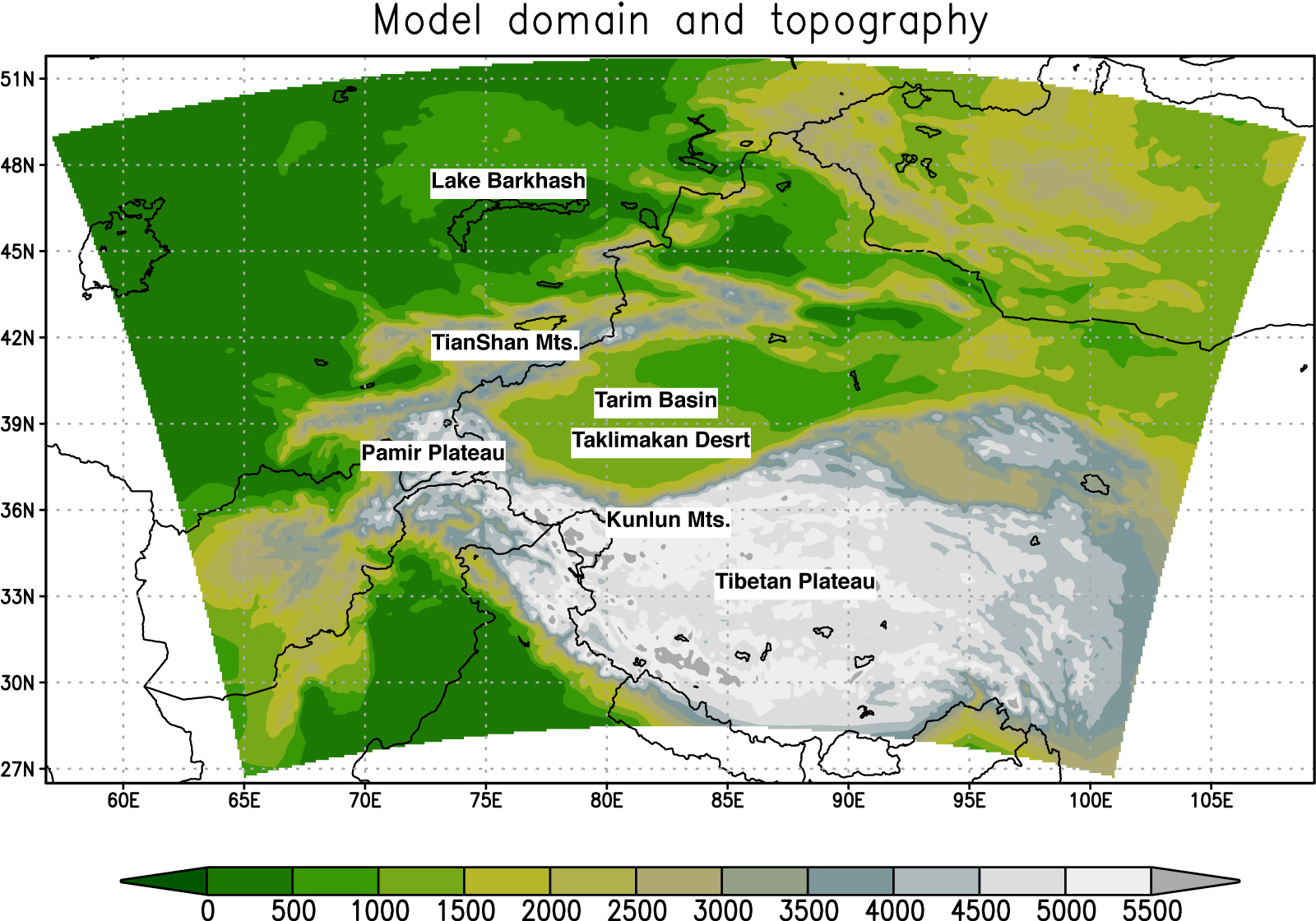


Fig. S1 The NHM-Chem modeling domain and topography of the Taklimakan Desert.


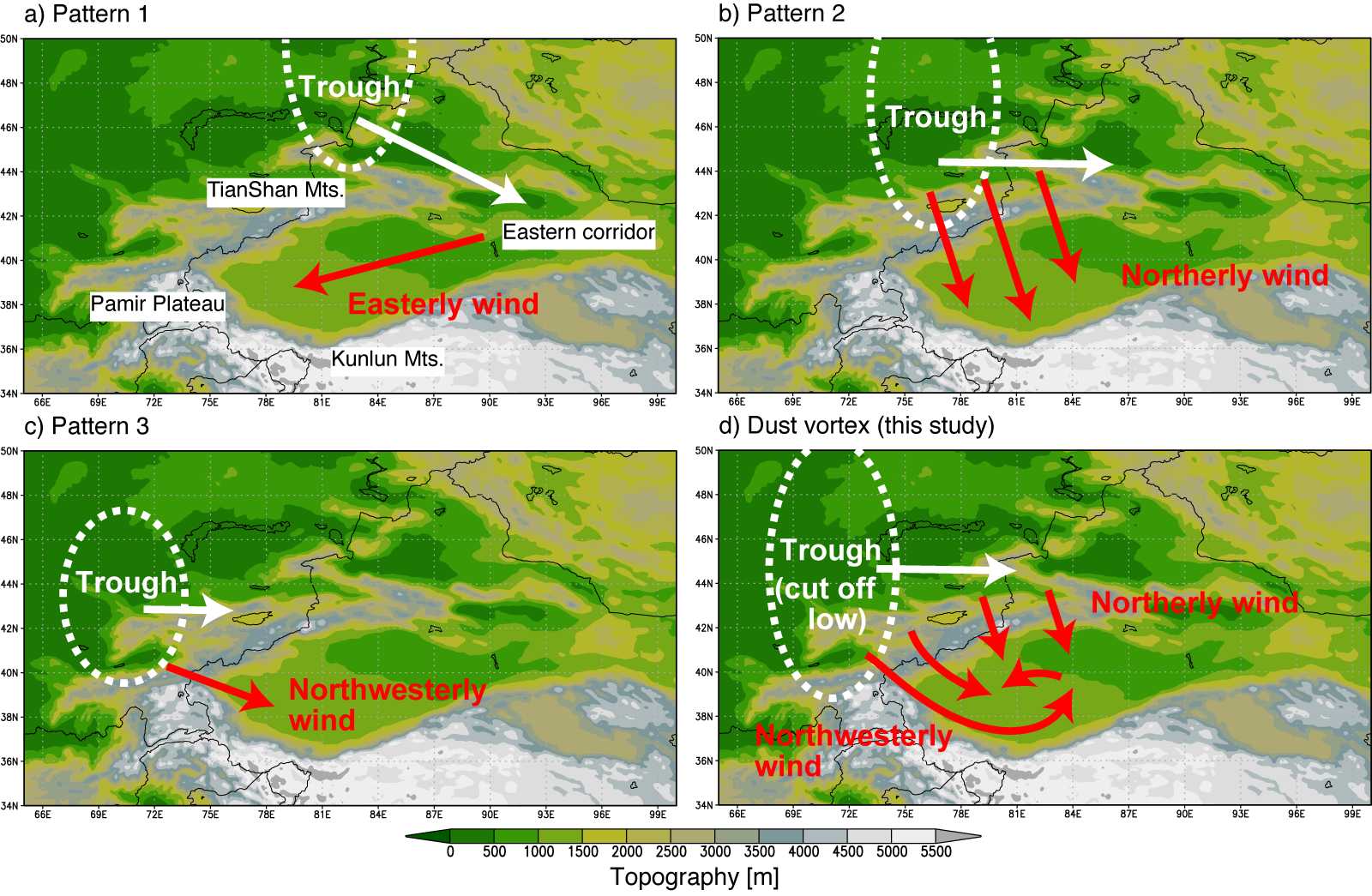


Fig. S2 Meteorological conditions and wind fields in the Taklimakan Desert for a) Pattern 1, b) Pattern 2, and c) Pattern 3, as classified by previous studies^1,2^. d) Meteorological conditions and wind fields of the dust vortex.


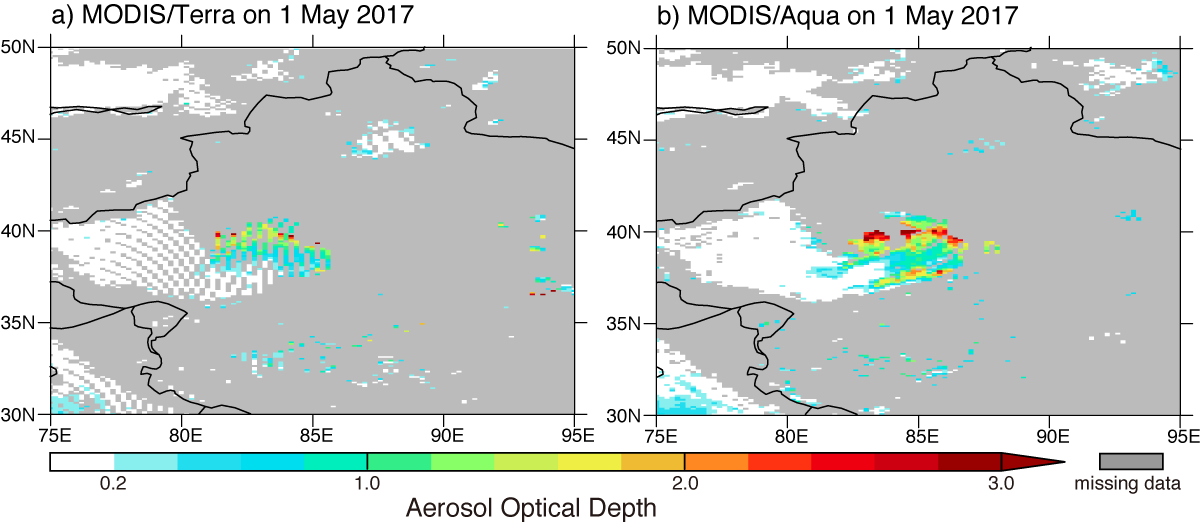


Fig. S3 Horizontal distributions of aerosol optical depth (AOD) provided by MODIS on board the Terra and Aqua satellites. We used AOD retrieved by the Deep Blue algorithm in the Level 2 correction 6 MODIS aerosol product (MOD04_L2.006 and MYD04_L2.006).


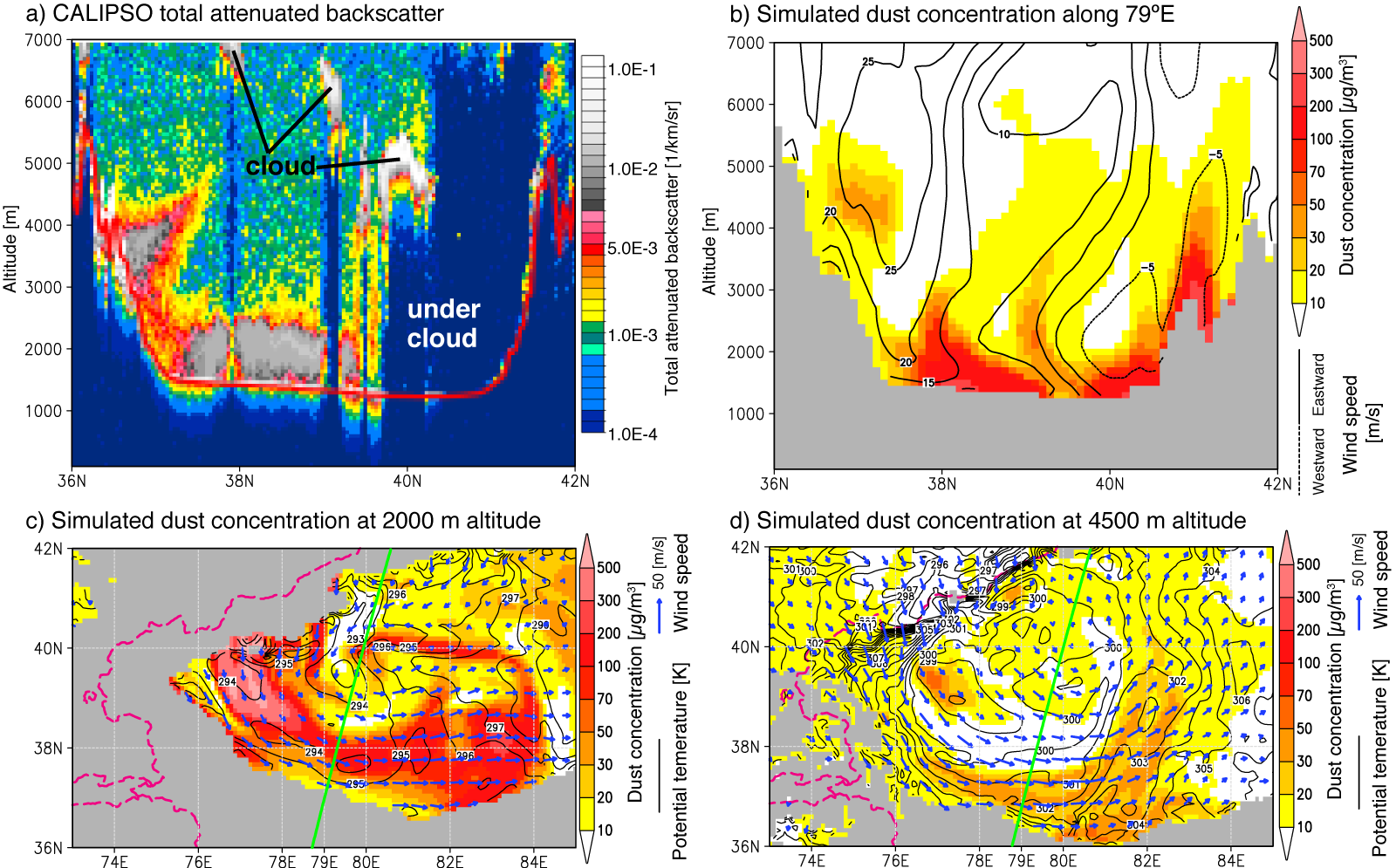


Fig. S4 CALIOP observations and NHM-Chem simulation at 21 UTC on 30 April. (a) Cross-sections of the CALIOP 532 nm total attenuated backscatter provided by the CAL_LID_L1-Standard-V4-10 (Level 1B, version 4.10) product (path# 2017-04-30T20-46-04ZN), (b) cross-section of the simulated dust concentration (color) and wind (black lines) in the east-west direction along 79°N, and (c–d) simulated dust concentrations (color), potential temperatures (black solid line), and horizontal wind fields (blue vectors) at 2000 and 4500 m altitudes. The green lines in (c–d) represent the CALIOP orbit path. Wind vectors are illustrated at 5 model-grid intervals.


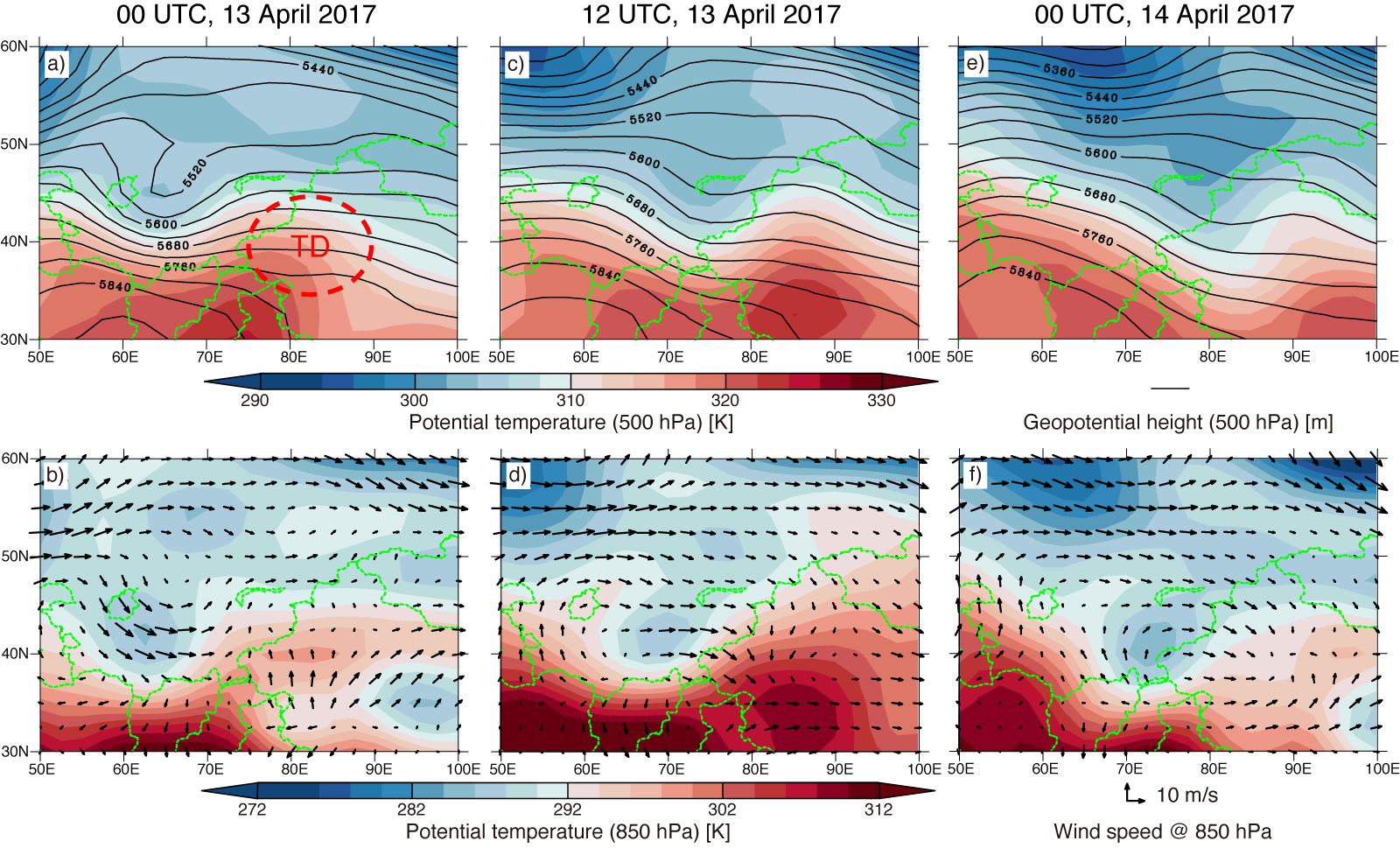


Fig. S5 Same as Figure 4, but for the dust vortex event during 13–14 April 2017.

**Supplemental Movie Legends**

**Movie S1.** Animation of the horizontal distribution of the Dust RGB imagery derived from Himawari-8 over the Taklimakan Desert (34°N–45°N, 70°E–95°E) during the dust vortex event on 30 April 2017 at 10-min intervals. In the Dust RGB image, airborne dust and clouds are depicted in magenta–pink and tan–brown, respectively. The Dust RGB images were obtained from “Himawari Real-Time Image/Central Asia/Dust RGB” (Meteorological Satellite Center of Japan Meteorological Agency) (http://www.data.jma.go.jp/mscweb/data/himawari/).

**Movie S2.** Animation of the horizontal distribution of the dust column load (magenta) simulated by NHM-Chem with simulated surface wind (black vectors) at 1-hour intervals. The region shown is the same as that in Movie S1. Green lines denote the topography. Wind vectors are illustrated at 5 model-grid intervals.

**Movie S3.** Same as Movie S1, but during the dust vortex event on 13 April 2017.
